# A sensitive mass spectrometry platform identifies metabolic changes of life history traits in *C. elegans*

**DOI:** 10.1038/s41598-017-02539-w

**Published:** 2017-05-25

**Authors:** Arwen W. Gao, Iliana A. Chatzispyrou, Rashmi Kamble, Yasmine J. Liu, Katharina Herzog, Reuben L. Smith, Henk van Lenthe, Martin A. T. Vervaart, Arno van Cruchten, Angela C. Luyf, Antoine van Kampen, Mia L. Pras-Raves, Frédéric M. Vaz, Riekelt H. Houtkooper

**Affiliations:** 1 Laboratory Genetic Metabolic Diseases, Academic Medical Center, 1105 AZ Amsterdam, The Netherlands; 2 Department of Clinical Epidemiology, Biostatistics and Bioinformatics, Academic Medical Center, 1105 AZ Amsterdam, The Netherlands

## Abstract

Abnormal nutrient metabolism is a hallmark of aging, and the underlying genetic and nutritional framework is rapidly being uncovered, particularly using *C. elegans* as a model. However, the direct metabolic consequences of perturbations in life history of *C. elegans* remain to be clarified. Based on recent advances in the metabolomics field, we optimized and validated a sensitive mass spectrometry (MS) platform for identification of major metabolite classes in worms and applied it to study age and diet related changes. Using this platform that allowed detection of over 600 metabolites in a sample of 2500 worms, we observed marked changes in fatty acids, amino acids and phospholipids during worm life history, which were independent from the germ-line. Worms underwent a striking shift in lipid metabolism after early adulthood that was at least partly controlled by the metabolic regulator *AAK-2*/AMPK. Most amino acids peaked during development, except aspartic acid and glycine, which accumulated in aged worms. Dietary intervention also influenced worm metabolite profiles and the regulation was highly specific depending on the metabolite class. Altogether, these MS-based methods are powerful tools to perform worm metabolomics for aging and metabolism-oriented studies.

## Introduction

Aging was long considered a passive process that could not be reversed. Over the recent decades, prolonged life expectancy in economically developed countries is proposed as a positive outcome of progress in medicine and modern lifestyle. It has become apparent that aging is amenable to treatment, either by physiological or pharmacological interventions, many of which directly or indirectly involve metabolic signaling pathways that interact in tightly controlled networks^1^. The involvement of nutrient sensing and defective mitochondria—two of the classical hallmarks of aging^2^—is emphasized by the fact that various mutations in metabolic genes extend lifespan^3^. Nutritional challenges and specific dietary interventions can also influence lifespan^4^; malnutrition is associated with short lifespan, while healthy nutrition promotes long life and healthy aging^5^. A prototypical example is caloric restriction, a condition of 20–50% reduced caloric intake that extends lifespan across species^6^. Furthermore, several studies in the nematode *Caenorhabditis elegans* showed that developmental rate, reproduction and lifespan were influenced when worms were fed with different bacterial strains^7–9^. Together, these studies accentuate the central role of metabolism in controlling animal lifespan.

To better understand the mechanistic underpinnings of metabolic changes during aging, metabolomics strategies were employed to measure the metabolic state in both mouse and human samples, which displayed marked changes in amino acid (AA) and fatty acid (FA) levels with age^10, 11^. For instance, aged mice were shown to have increased free FA levels in plasma compared to young mice, while intermediate metabolites of FA oxidation and AAs were decreased with old age^10^. Measuring a variety of metabolites allows exploration of the specific metabolic derangements that occur in the aging individual, and can serve as biomarkers for aging and healthspan. On the other hand, mammalian models are not optimal for establishing the role of gene networks in metabolism and aging, as genetic modification is difficult to achieve in a large-scale approach. *C. elegans* is one of the most popular models to investigate the networks that govern aging pathways, due to (1) its relatively short lifespan, (2) its fully sequenced genome, and (3) the ease of genetic intervention with RNAi through bacterial feeding. The impact of nutrition and metabolism on *C. elegans* aging is evident from various studies. Genetic intervention studies demonstrated the involvement of metabolic pathways, e.g. proline metabolism, in the regulation of aging in the long-lived *daf-2* (insulin receptor) mutant worms, as well as in worms exposed to different bacterial strains as dietary sources^8, 12^. In order to quantify the effects of metabolic interventions, metabolite measurements are commonplace in mammalian studies but are still limited for *C. elegans*, where they have only recently started to be applied more extensively^13^. Amongst the most commonly used methods to perform metabolomics in worms are those based on gas chromatography-mass spectrometry (GC-MS)^14–16^ and nuclear magnetic resonance (NMR) spectroscopy^13, 17^.

In order to dissect age and diet-related changes in the *C. elegans* metabolome, and bearing in mind the current advances in the field, we set out to validate a sensitive MS-based platform that would allow us to further expand the spectrum of detectable metabolites in *C. elegans*, without compromising accuracy in quantification and while retaining sample size to the minimum. We first optimized and validated methods for the measurement of FAs, AAs and phospholipids (PLs)—three major metabolite classes—totaling over 600 metabolites in a sample of approximately 2500 worms. Next, we applied our platform to identify changes during physiologically relevant metabolic states. We analyzed metabolite profiles during life history, including larval development, the reproductive phase, and aging. In addition to life history traits, we also investigated how feeding worms on different bacterial strains alters worm metabolite profiles. Altogether, our data highlight how application of metabolomics is a powerful tool to uncover mechanisms that underlie the link between metabolic changes and aging in *C. elegans*.

## Results

### Development of fatty acid and amino acid analyses in *C. elegans*

**Figure 1:**
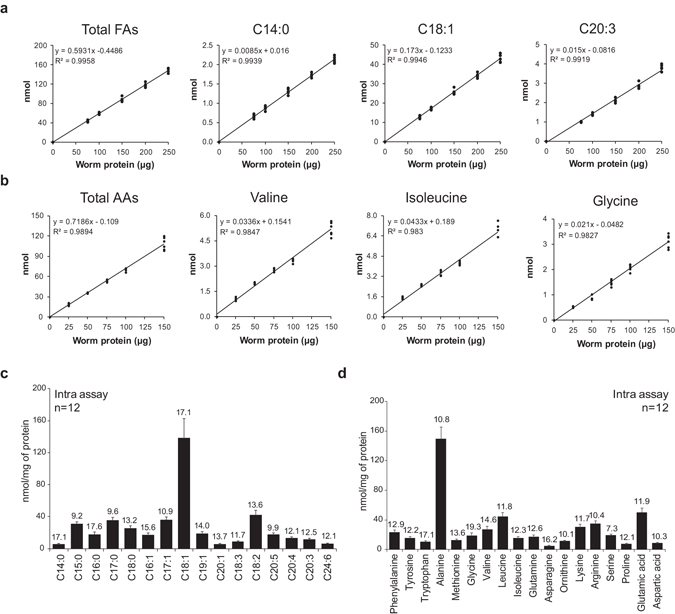
Linearity and precision of fatty acid (FA) and amino acid (AA) analysis in *C. elegans*. (**a**) FAs were extracted from increasing amount of worm protein lysate (0–250 µg) and measured using MS. A total of 35 FAs were detectable. A subset of FAs is shown here. (**b**) AAs were extracted from 0–150 µg of worm protein lysate and measured using UPLC-MS/MS. Eighteen AAs were detected and considered linear. A subset of AAs is shown here. (**c**,**d**) Twelve identical biological samples were independently extracted and measured within the same analytical run to determine intra-assay (within day variation) variation for FAs (**c**) and AAs (**d**). Bar graphs are expressed as mean ± SD. Values above the bars indicate the coefficient of variation. See also Supplementary Fig. S1 and Supplementary Table S1.

To study metabolic changes of life history traits and diet interventions in *C. elegans* in depth and with high accuracy, we established sensitive MS methods for *C. elegans*, based on methods used for human and murine tissues. For the measurement of FAs we applied MS, and for AAs we used UPLC-MS/MS^18–20^. We first determined the amount of worm lysate that was required to measure FAs and AAs, and whether measurement and quantification was linear with the amount of extracted material. FAs were extracted from worm lysates containing 0 to 250 µg of protein (Fig. 1a; Supplementary Fig. S1a), and AAs were extracted from worm lysates containing 0 to 150 µg of protein (Fig. 1b; Supplementary Fig. S1b). We detected 44 medium-chain, long-chain, and very-long-chain FAs (C14:0 to C30:0), as well as 18 different AAs (Fig. 1; Supplementary Fig. S1 and Supplementary Table S1). Both metabolite classes showed linear responses for the entire range of protein extracted, and could even be detected in quantities as low as 75 µg of worm protein lysate for FA species and 25 µg for AAs. We also determined the limit of detection (LOD) and limit of quantification (LOQ) for both metabolite classes. For FA, the LOD was between 0.006–0.01 nmol/mg protein, and the LOQ was 0.02–0.03 nmol/mg protein, allowing quantification of 35 FA species; for AA, the LOD was between 0.1–2 nmol/mg protein and the LOQ was 0.3–4 nmol/mg protein. Taking into consideration both the LOD/LOQ and the ease of preparing worm samples, we chose to continue our validation with 150 µg of protein for FA and 50 µg for AA, an amount that corresponds to approximately 500 worms and allows extraction of both metabolite classes from the same lysate.

To establish the precision of the FA and AA analyses, we determined the variation in three ways. First, we checked the MS technical variation, in which a single sample was injected 21 times into the MS apparatus. The MS variation was between 0.6–2.5% for FA measurements (Supplementary Fig. S1c and Supplementary Table S1) and between 1.4–7.7% for AA measurements (Supplementary Fig. S1d and Supplementary Table S1). Second, we determined the intra-assay (within day) variation of the FA and AA analyses in identical samples that were extracted and measured independently within the same analytical run. We found that the intra-assay variation of FA analysis was between 9.2–17.6%, (Fig. 1c; Supplementary Table S1), and the variation of AA analysis was between 7.3–19.3% (Fig. 1d; Supplementary Table S1). This indicates that the variation caused during the extraction procedure was limited in most analytes except for a few FA and AA species that have low abundance in worms. Third, we determined the inter-assay (day-to-day) variation by extracting and measuring FAs and AAs from identical samples on different days and analytical runs. The inter-assay variation of both the FA and AA analysis was very similar to the intra-assay variation, confirming the precision of our platform (Supplementary Fig. S1e,f and Supplementary Table S1).

### Validation of FA and AA analyses in worms with deficient lipid or AA metabolism

**Figure 2:**
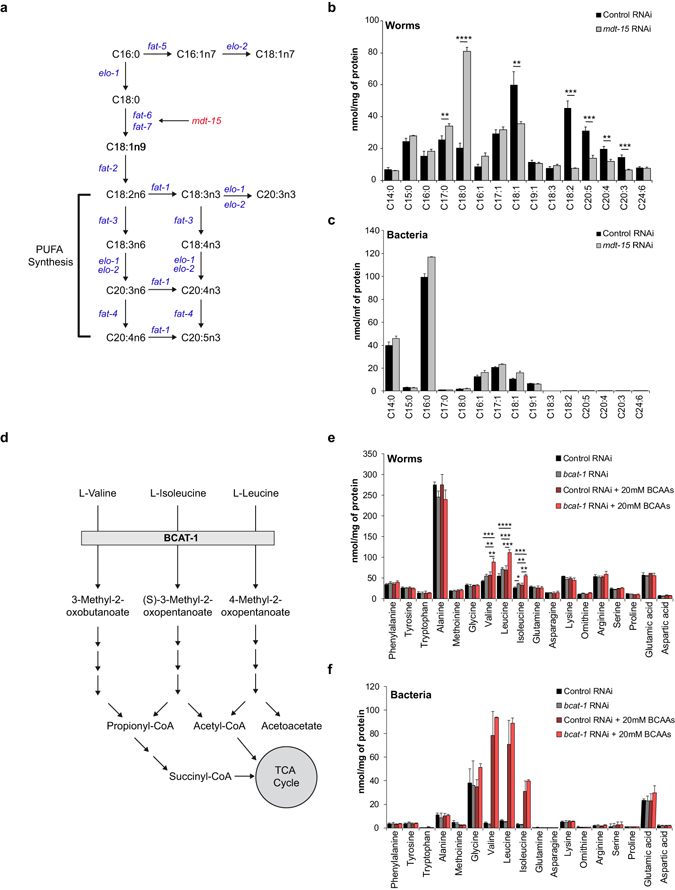
Validation of FA and AA analysis in *C. elegans* with deficient lipid or AA metabolism. (**a**) Schematic representation of polyunsaturated fatty acid (PUFA) synthesis pathway in *C. elegans*. *mdt-15* is a key regulator in this pathway that controls the activity of stearoyl-CoA desaturases *fat-6* and *fat-7*. (**b**) N2 worms exposed to *mdt-15* RNAi accumulated C18:0 and had reduced levels of C18:1 and PUFAs. Bar graphs are expressed as mean ± SD; Significance was calculated using Student’s *t*-test. *p ≤ 0.05; **p ≤ 0.01; ***p ≤ 0.001; ****p ≤ 0.0001. (**c**) FA composition in *mdt-15* RNAi bacteria was similar to that in the control RNAi bacteria. No PUFAs were detected in either RNAi bacteria. (**d**) Schematic representation of branched-chain amino acid (BCAA) catabolism. Branched-chain amino acid aminotransferase 1 (*bcat-1*) catalyzes the first reaction of this pathway. (**e**) Accumulation of BCAAs valine, leucine and isoleucine was detected in *rrf-3(pk1426)* worms treated with *bcat-1* RNAi. Worms grown on plates supplemented with 20 mM BCAAs accumulated BCAAs, which was more pronounced in combination with *bcat-1* RNAi. Bar graphs are expressed as mean ± SD, Significance was calculated using One-way ANOVA; *p ≤ 0.05; **p ≤ 0.01; ***p ≤ 0.001; ****p ≤ 0.0001. (**f**) BCAAs levels were similar in control and *bcat-1* RNAi bacteria, but accumulated in BCAA-supplemented bacteria. See also Supplementary Fig. S2.

To confirm that we can reliably measure relevant changes *in vivo*, we applied our metabolomics platform to analyze changes in FA and AA profiles in worms with a deficiency in lipid or amino acid metabolism, respectively. First, we determined the FA composition in *mdt-15* and *fat-7* RNAi worms. The *mdt-15* gene encodes a subunit of the Mediator complex that is orthologous to mammalian MED15 and is an essential regulator of lipid metabolism in *C. elegans* (Fig. 2a)^16, 21^. MDT-15 controls the expression of FA desaturase genes including *fat-6* and *fat-7* and is required for the synthesis of polyunsaturated fatty acids (PUFAs)^21^. Indeed, by applying our method we detected a marked increase of C18:0 in *mdt-15* RNAi worms when compared to worms fed with control RNAi, while C18:1 levels were decreased (Fig. 2b). Since C18:1 is the precursor for the synthesis of PUFAs^16^, *mdt-15* RNAi also led to markedly reduced levels of PUFA species, such as C18:2, C20:3, C20:4 and C20:5 (Fig. 2b). These findings are in line with previous observations, therefore confirming our method’s accuracy^21^. In order to exclude that the FA alterations are due to unexpected changes in the bacterial FA composition, we also measured the FA composition in the *E. coli* HT115 bacteria that were plated for the same time period without worms. The FA composition in *mdt-15* RNAi bacteria was similar to that in the control RNAi bacteria (Fig. 2c), confirming that the differences in FA composition were strictly due to the knockdown of *mdt-15*. Similar changes in the FA compositions were detected in *fat-7* RNAi worms (Supplementary Fig. S2a,b).

To validate the method for AA analysis, we measured the AA profile of worms deficient in *bcat-1*, which encodes a branched-chain amino acid aminotransferase that catalyzes the first reaction in branched-chain amino acid (BCAA) catabolism^22, 23^ (Fig. 2d). Consistent with this, the BCAAs valine, leucine, and isoleucine accumulated in *bcat-1* RNAi worms compared to the control (Fig. 2e), leading to a 25.3% higher ratio of BCAAs over total AAs (BCAAs/total AAs) (Supplementary Fig. S2c). We then supplemented 20 mM BCAAs in the culture medium to increase the pressure on BCAA catabolism. We found an overall accumulation of BCAAs in both *bcat-1* RNAi and control worms grown on plates supplemented with 20 mM BCAAs compared to those grown on regular plates (Fig. 2e); the BCAAs/total AA ratio was 19.7% higher in control worms supplemented with 20 mM BCAAs, and the ratio further increased by 33.1% when *bcat-1* RNAi was combined with BCAA supplementation (Supplementary Fig. S2c). These changes were not caused by the bacterial food source, as bacterial BCAA levels were similar in both the control and *bcat-1* RNAi condition (Fig. 2f; Supplementary Fig. S2c). Our observations of BCAA accumulation in *bcat-1* RNAi worms are in line with previous work^24^, confirming the validity of our method.

### Development and validation of phospholipid analysis

Further on, we set out to measure phospholipids (PLs) in *C. elegans* using HPLC-MS, thus expanding the spectrum of detectable metabolites in worms. We based our method on a previously described lipidomics approach for human fibroblasts and tissues^25, 26^ that allowed accurate detection for a large number of different PL species in human samples, which we adapted and optimized for worm preparations. We first determined the linearity of PL analysis by extracting and measuring PLs from increasing numbers of worms (0, 500, 1000, 2000 and 4000 worms). We determined protein content in each sample, and normalized the relative PL abundance to both number of worms and protein content to ensure reproducibility of the worm recovery from the plates. We then correlated the abundance of each metabolite to both the amount of worms and protein content. In all samples, even with lower numbers of worms, we were able to measure ~600 PL species (Supplementary Table S1). We observed that the number of worms as well as the protein content of the samples correlated linearly with the relative abundance of the majority of detected PLs (Supplementary Table S1). Based on these results, we chose to continue our PL analyses using 2000 worms, since it was well within the linear range for most metabolites and because of the ease of sample preparation.

**Figure 3:**
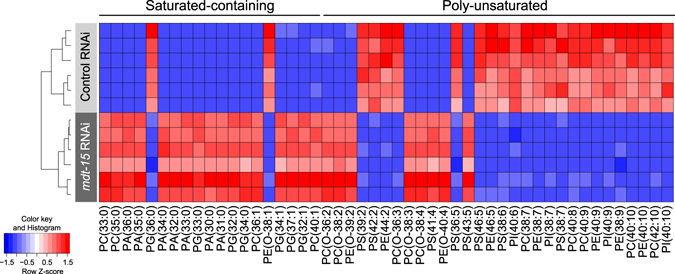
Phospholipid (PL) profiles in *C. elegans* after *mdt-15* RNAi. Unsupervised hierarchical clustering of PL composition in N2 worms treated with *mdt-15* RNAi. After *mdt-15* RNAi, worms accumulated PLs with saturated acyl chains, and had decreased levels of PL species with a high degree of unsaturation (≥6 double bonds). Abundance of PLs with “intermediate” degree of saturation (1–5 double bonds) did not present a standard pattern between the *mdt-15*-treated and control worms. Phospholipids are designated as C(XX:Y), where XX denotes the total number of carbon atoms and Y the total number of double bonds in the fatty acyl chains. See also Supplementary Table S1.

Next, in order to validate our method, we analyzed the PL composition in worms fed with *mdt-15* RNAi, which has been previously been shown to be shifted towards a lower degree of unsaturation^27^. We employed principal component analysis (PCA) to identify the PLs that contribute most to the clustering of the samples in terms of differential abundance. The PCA allowed us to clearly distinguish worms treated with *mdt-15* RNAi from those treated with control RNAi (HT115) (data not shown). When applying hierarchical clustering on all samples, the control and *mdt-15* RNAi-treated groups were separated in an unbiased manner (Fig. 3). Similar to the changes found in FA composition (Fig. 2b), worms treated with *mdt-15* RNAi accumulated PL species with saturated acyl chains (Fig. 3). Conversely, PL species containing acyl chains with a high degree of unsaturation—with ≥6 double bonds, i.e. containing PUFAs—were markedly decreased in these worms (Fig. 3). While the overall profile confirms the expected shift in FA synthesis, there are some apparent exceptions. For instance, despite the overall switch to saturated lipids in the *mdt-15* RNAi condition, some PL species containing 1–5 double bonds (e.g. PC(O-38:3), PC(O-38:4), PS(41:4), PE(O-40:4) PS(43:5)), were increased in *mdt-15* RNAi worms. Furthermore, we observed a marked decrease in PG(36:0) in *mdt-15* RNAi worms. This profile is opposite to the other saturated species, but since *mdt-15* RNAi worms can normally synthesize saturated species (Fig. 2b), it rather suggests that the abundance of this lipid is regulated. Together, our observations confirmed that the PL method is sensitive and allows the detection of changes in lipid metabolism.

### Metabolic changes in *C. elegans* life history

**Figure 4:**
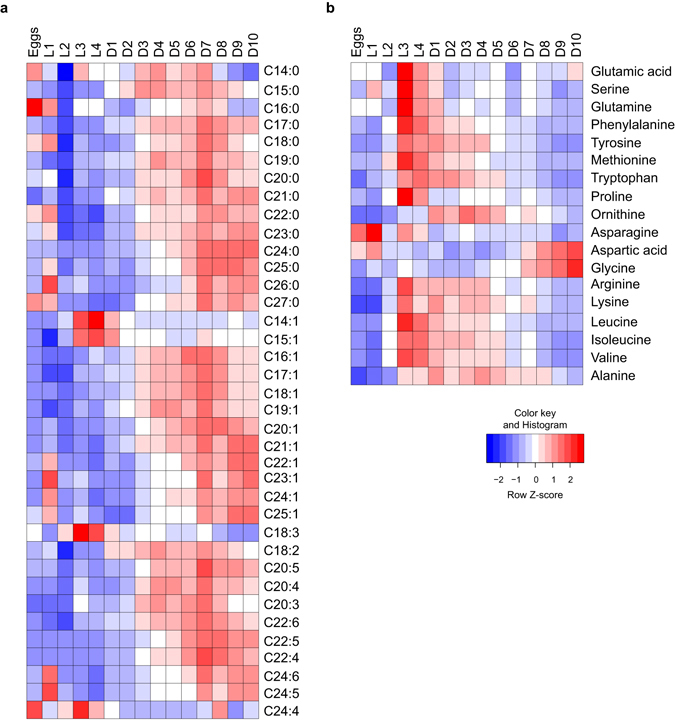
FA and AA changes during life history of *C. elegans*. (**a**) FAs were measured in the different life history stages of *C. elegans*, i.e. eggs, four larval stages, and the first 10 days of adulthood. The abundance of FAs was low during the larval stages, increased during the reproductive phase (day 2–5) and decreased post-reproduction after reaching a peak at day 7 of adulthood. C18:3 reached the highest abundance during the later developmental phase and decreased with age. (**b**) Most AAs reached a peak during the later larval stages and decreased in the adult phase. The abundance of aspartic acid and glycine remained low in development and early adulthood, and significantly increased at later ages. See also Supplementary Fig. S3.

Because we aim to study how metabolite composition changes in the aging process, we applied our metabolomics platforms to characterize the metabolic changes of life history traits in *C. elegans*. We collected worms at different stages of life history, including eggs, four larval developmental phases (L1-L4), the reproductive phase (day 1–5 of adulthood) and the aging phase (day 5–10 of adulthood). The abundance of most FA species was low during the larval development stages, and increased in the reproductive phase of adult life (Fig. 4a; Supplementary Fig. S3a). Most FAs reached a peak around the post-reproductive day 7 of adulthood and slowly declined until day 10 (Fig. 4a; Supplementary Fig. S3a). Some FA species, however, showed a markedly different profile during development and aging. For instance, C14:1, C15:1 and C18:3 reached the highest abundance during the later development phase and decreased with age. On the other hand, the very-long-chain FAs C24:0, C21:1 and C22:1 reached the highest levels at day 10 (Fig. 4a; Supplementary Fig. S3a), indicating that these FAs accumulate with age.

In contrast to the changes found in FA composition, most AA species reached the highest abundance during the later larval stage and early adulthood, and decreased throughout the adult phase (Fig. 4b; Supplementary S3b). Some AA species, however, followed a different profile; serine, proline, glutamine, and glutamic acid reached their peak abundance during the later larval stages, and then dropped to a steady level at early adulthood that was maintained throughout life (Fig. 4b; Supplementary Fig. S3b). The abundance of asparagine was high in worm eggs and the first larval stage (L1), and then declined throughout larval and adult stages. Finally, the abundance of glycine and aspartic acid remained low in development and early adulthood, but continued to increase until day 10 (Fig. 4b; Supplementary Fig. S3b).

**Figure 5:**
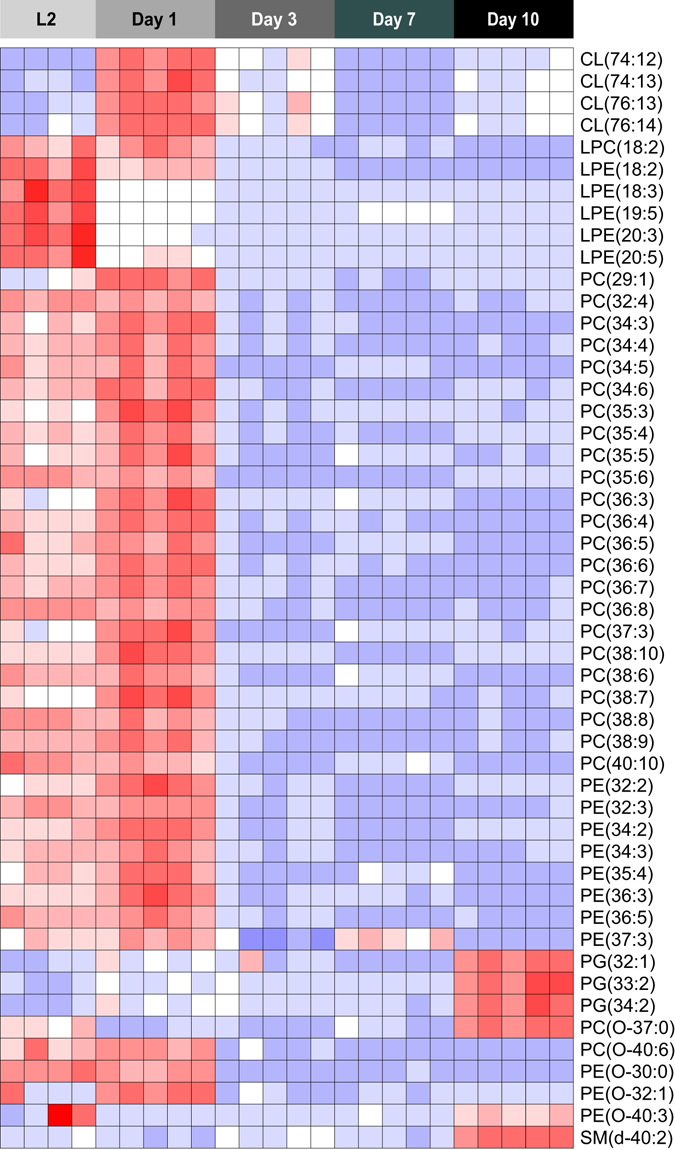
PL profiles during life history of *C. elegans*. PLs were measured in different stages of *C. elegans* life history. Several PL species, including many PCs, PEs, and LPEs were higher in L2 larvae and day 1 adults, and declined with age. Some CL species displayed a peak on day 1, while remaining at lower abundance during other life stages. Conversely, a few PG species had low abundance in early and mid- life stages, and displayed a marked increase in day 10 adults. See also Supplementary Table S1.

In the case of PLs, we first identified which of the ~600 identified species contribute most to the separation of young versus aged, by employing PCA analysis between the day 1 and day 10 worms (data not shown). When looking at the abundance profiles of these metabolites in different phases of the worm life history, we observed that some particular PL species, such as many phosphatidylcholines (PCs) and phosphatidylethanolamines (PEs), were abundant during the larval stage and early adulthood (day 1) and lowered during the remainder of adult lifespan (Fig. 5). Lysophosphatidylethanolamines (LPEs) followed a similar pattern, but with a more pronounced increase during the L2 larval stage, while some cardiolipins (CLs) presented low abundance in L2 stage and a peak during early adulthood. Other PLs such as phosphatidylglycerol (PG) and a sphingomyelin (SM) species presented an opposite pattern; they were less abundant during the larval and early adult stages and accumulated at a later stage of life (day 10) (Fig. 5).

### Determinants of age-related metabolic changes

**Figure 6:**
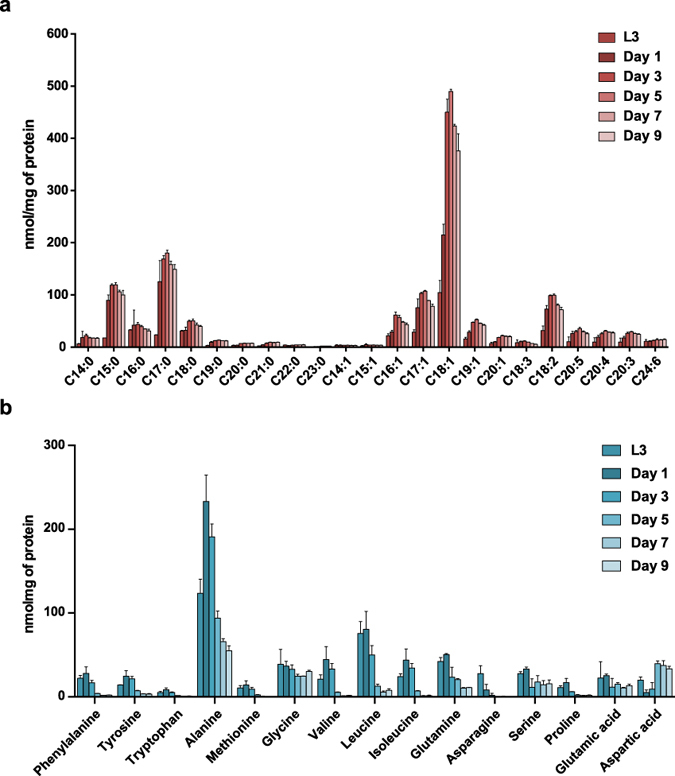
Metabolite changes in *glp-4*(*bn2*) mutant worms during life history. (**a**) FAs were measured in different ages of *glp-4*(*bn2*) mutants, including the larval stage 3 (L3), the reproductive phase (day 1–5 of adulthood), and post-reproductive phase (day 7–9). Most FA species increased during adult life, and slightly decreased in the post-reproductive phase. C18:3 reached its highest abundance at younger stage then decreased with age. (**b**) Most AA species reached a peak at L3 and day 1 of adulthood and subsequently decreased with age in *glp-4*(*bn2*) mutant worms. In the *glp-4*(*bn2*) worms aspartic acid accumulated and glycine remained more or less equal, similar to the changes observed in the wild type N2 worms grown under the same culture conditions (25 °C and without 5FU). See also Supplementary Fig. S4.

To start dissecting the possible impact of the germline on the metabolic changes we observed in worms during the course of life history (Fig. 4), we also profiled *glp-4*(*bn2*) mutant worms, that are deficient in germline cell proliferation and gametes formation when grown at 25 °C^28^. We collected *glp-4*(*bn2*) mutants at larval stage L3, reproductive phases of adulthood on day 1, day 3 and day 5, and elderly stages day 7 and day 9 and measured FAs and AAs. Similar to what we observed in the FA profiles of N2 worms during life history, most FAs accumulated with age in the *glp-4*(*bn2*) mutants and followed a small decrease in late adulthood (Fig. 6a, Supplementary Fig. S4a). Some FAs showed a different profile during development and aging, which was consistent with the exceptions observed in the N2 worms; C14:1 and C15:1 reached their highest level at young age and remained at lower levels in aged worms while C18:3 reached the highest abundance in young worms and gradually decreased with age.

For AAs, the pattern of abundance during life history was identical between *glp-4*(*bn2*) mutants and N2 worms (Fig. 6b, Supplementary Fig. S4b); most AAs reached their peak abundance early in life and decreased with age with glycine and aspartic acid being notable exceptions (Fig. 6b, Supplementary Fig. S4b). It is worth noting, however, that the profiles of the latter two AAs followed slightly different patterns under the conditions employed for the *glp-4*(*bn2*) mutant than under standard *C. elegans* culture conditions. Under standard conditions, i.e. cultured at 20 °C and in the presence of 5-fluorouracil (5FU), glycine and aspartic acid continued to accumulate with age (Fig. 4b). At 25 °C and without 5FU, aspartic acid levels were high in L3 larvae, lowered at the first days of adulthood, and then increased with age; glycine levels remained more or less stable throughout life. The same pattern was observed also for the N2 worms cultured at 25 °C as a control (Supplementary Fig. S4b). Whether these changes were caused by culturing at higher temperature or because of interference of 5FU is at present unclear, but considering that both FA and AA profiles were similar between wild type N2 worms and the *glp-4*(*bn2*) mutant we conclude that the metabolite changes identified in *C. elegans* life history are independent of the germline.

**Figure 7:**
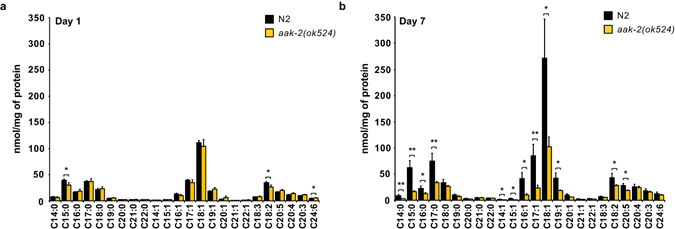
Metabolite profiles in young and aged *aak-2* mutants. (**a**) In early adulthood (day 1), the abundance of FAs in *aak-2*(*ok524*) was similar to N2 worms. (**b**) While most FAs accumulated in aged N2 worms (day 7), this accumulation was attenuated in aged *aak-2*(*ok524*) worms, suggesting that this gene drives the observed age-related changes in lipid metabolism. Bar graphs are expressed as mean ± SD, Significance was calculated using Student’s *t*-test. *p ≤ 0.05; **p ≤ 0.01; ***p ≤ 0.001; ****p ≤ 0.0001.

Following our striking observations in FA profiles throughout *C. elegans* life history, we next aimed to identify which metabolic regulator drives the age-related changes in lipid metabolism, and hypothesized that the key signaling hub *aak-2* (AMPK) would be involved considering its role in managing mitochondrial metabolism^29, 30^. We therefore set out to compare FA levels between *aak-2* mutants and N2 wild type worms in a time-resolved manner, using samples from young (day 1) and aged (day 7) worms, the latter representing the peak of lipid abundance in wild type worms (Fig. 4a). While at day 1 the FA levels were similar between the two groups, at day 7 the observed lipid accumulation was strongly attenuated in the *aak-2* mutants (Fig. 7a,b). Together, these data suggest that *AAK-2* drives the observed changes in lipid metabolism in aging.

### The effect of diet on worm metabolite profiles

**Figure 8:**
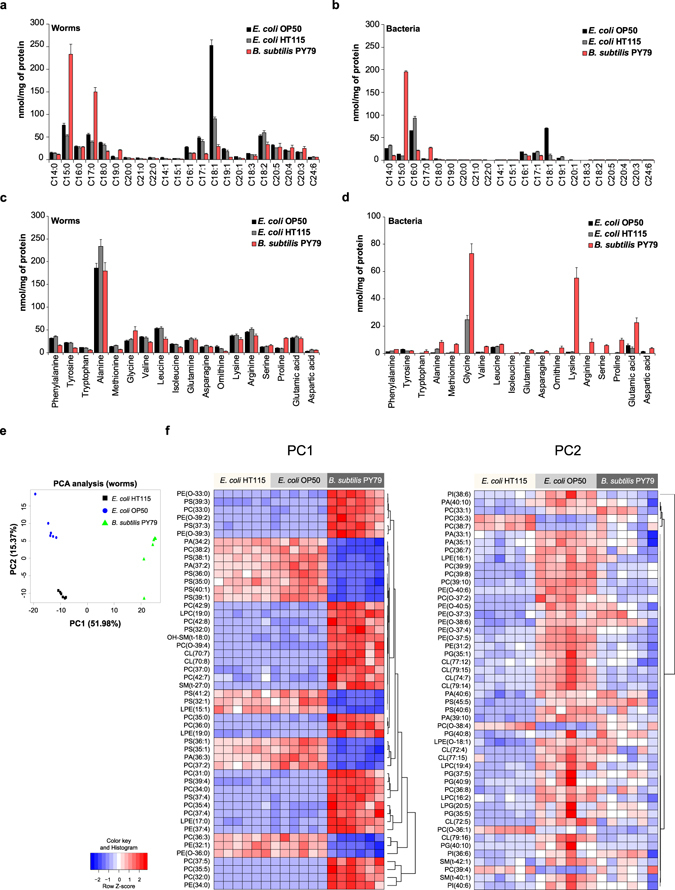
Metabolite changes in worms exposed to different bacterial diets. The effect of dietary intervention on worm metabolism was investigated by feeding N2 worms with three types of bacteria including *E. coli* OP50, *E. coli* HT115 and *B. subtilis* PY79. (**a**) FA composition of N2 worms fed with different bacterial diets. Worms fed an *E. coli* OP50 diet accumulated the highest abundance of C18:1 fatty acid. A relatively high abundance of C18:1 was also found in worms fed with the other *E. coli* strain HT115. Worms fed a *B. subtilis* PY79 diet accumulated odd chain fatty acids, such as C15:0 and C17:0. (**b**) FA composition of the three bacterial diets. The two *E. coli* bacterial strains were enriched with C14:0, C16:0 and C18:1, while *B. subtilis* PY79 bacteria were enriched with odd chain fatty acids C15:0 and C17:0. Polyunsaturated FAs were absent in all three bacterial strains. (**c**) AA composition of N2 worms fed with different bacterial diets. AA profiles of worms fed with different bacterial diets did not display marked changes. Worms fed the *E. coli* strains showed similar AA profiles, while feeding worms with a *B. subtilis* diet showed a high abundance of proline. (**d**) AA composition of the three bacterial strains. *E. coli* strains contained low levels of most AA species, except glycine, which is relatively high abundant in *E. coli* HT115. *B. subtilis* PY79 bacteria were enriched in many AA species, especially glycine, lysine and glutamic acid. (**e**) Principal Component Analysis (PCA) score plot showing group separation based on PL profiles in N2 worms fed with different bacterial diets. Worms fed a *B. subtilis* PY79 diet were clearly separated from those fed with an *E. coli* diet, and a distinctive separation was evident between worms fed OP50 or HT115 *E. coli*. (**f**) PL composition in N2 worms fed with different bacterial diets. The metabolites contributing most to the groups’ separation based on PC1 and PC2 of the PCA are shown. Bar graphs are expressed as mean ± SD. See also Supplementary Fig. S5 and Suplementary Table S1.

To explore the effect of dietary interventions on worm metabolism, we exposed N2 worms to different types of bacterial strains as their food, including the two *E. coli* strains OP50 and HT115, and the *Bacillus subtilis* strain PY79. We compared metabolite profiles in worms exposed to these diets to the metabolites in the bacteria themselves. Worms fed with different types of bacteria showed different FA and AA compositions. We observed a high abundance of C15:0 and C17:0 in worms fed with *B. subtilis* PY79 compared to worms fed with either of the two *E. coli* diets (Fig. 8a). The high levels of C15:0 and C17:0 reflected the levels observed in *B. subtilis* PY79 bacteria themselves when compared to the *E. coli* bacteria (Fig. 8b). Between the two *E. coli* strains, worms fed with an *E. coli* OP50 diet were markedly enriched with C18:1 compared to worms fed with *E. coli* HT115 (Fig. 8a). The FA composition in worms showed clear similarities with the *E. coli* they were fed on, as OP50 bacteria were also enriched with C18:1. Although most worm FAs reflect the bacteria they eat, some were absent in all three bacterial strains, particularly the PUFA species, confirming that these FAs are mainly synthesized *de novo* in *C. elegans* ^15^.

The AA composition was similar in worms fed with different bacterial diets (Fig. 8c), despite the fact that the metabolite composition of the bacteria themselves was markedly different (Fig. 8d). For instance, *B. subtilis* PY79 contained high levels of glycine, lysine, serine and glutamic acid, but these AAs were not enriched in the PY79-fed worms (Fig. 8c,d). The fact that very different bacterial AA profiles lead to almost identical worm AA composition suggests that the AA levels in worms are tightly controlled and subject to regulation.

In a similar fashion, we measured PLs in worms exposed to the three bacterial diets and in the bacteria themselves. PCA analysis of the different samples distinctively separated the worms exposed to *B. subtilis* PY79 from those exposed to the two *E. coli* strains, and also distinguished the different *E. coli* strain-fed worms from each other (Fig. 8e,f). The PL composition in the different bacterial diets was also very distinctive (Supplementary Fig. S5), but differed strikingly from the PLs identified in worms (Supplementary Table S1). Together, these data suggest that the diet influences the PL composition in worms. On the other hand, worm PL species do not necessarily reflect those found in the bacterial diets, but are the product of endogenous synthesis^13^, that utilizes the bacteria-derived building blocks.

Overall, our findings demonstrate that bacterial diets of different metabolite compositions can impact on the *C. elegans* metabolome, which may be a result of direct integration (FAs), tight regulation (AAs) or indirect use for anabolic processes (PLs).

## Discussion

Metabolic networks and nutrient sensing pathways play an important role in the aging process. Despite the progress in recent years on the elucidation of molecular actors in aging and longevity, many of the metabolic mechanisms are still poorly understood, including the interaction between genetic regulators and the environmental or nutritional exposure. In order to obtain information at a molecular level during aging and to elucidate the response of a living organism to nutrient stimuli, it is important to measure metabolites in aging model organisms such as *C. elegans*. Several significant steps were made in this direction using GC-MS^14, 15, 31^ and NMR^17, 32^. We built on this knowledge and validated a platform suitable for the identification and quantification of 35 FAs and 18 AAs in a sample that is equivalent to approximately 500 worms, and ~600 PLs in a sample of 2000 worms. We applied the platform to determine metabolic changes in development, reproduction and aging. We first optimized and validated the methods to measure FAs by MS, AAs by UPLC-MS/MS, and PLs by HPLC-MS in *C. elegans*. We further validated the methods by measuring FAs, PLs, and AAs in worms with a deficiency in PUFA synthesis or BCAA catabolism. We observed an accumulation of C18:0 and decreased levels of C18:1 and PUFAs in worms treated with *mdt-15* and *fat-7* RNAi^21^. The changes we observed at the level of FAs were also reflected in the acyl chain composition of PLs in worms fed with *mdt-15* RNAi; PLs with saturated acyl chains accumulated, while those with a high degree of unsaturation were decreased^27^. For the AA measurement, we observed BCAA accumulation in worms fed with *bcat-1* RNAi^24^, which was further exacerbated after BCAA supplementation. Interestingly, even though the bacteria were ~18x enriched in BCAA, the BCAA accumulation in worms increased only 2-fold, suggesting that AA uptake, distribution, and/or metabolism is tightly controlled. Together, these experiments strongly validate our metabolomics platform.

Next, we applied our platform to elucidate the metabolic changes of life history traits in *C. elegans*. Most FA species were in low abundance during development, and accumulated from day 3 to day 7 of adulthood. The overall increase in abundance of FAs in aged versus young worms is similar to previous observations in aged mice^10^, suggesting that changes in FA profiles in life history could be at least partly conserved among different model organisms. Notably, the age-related lipid accumulation we observed in wild type worms was attenuated in the *aak-2* mutants, suggesting involvement of this pathway in lipid regulation in aging. Moreover, the changes in FA levels during *C. elegans* life history were not related to the germline, since metabolic profiling of the sterile *glp-4*(*bn2*) mutants deficient in germ cell proliferation showed similar fluctuations to those observed in wild type worms.

Most AA species were more abundant during the later developmental stages and early adulthood, and decreased after day 4 of adulthood. In addition, we found a remarkable accumulation of aspartic acid and glycine in aged worms. Although future work will have to elucidate the mechanisms behind the accumulation, it is striking that these amino acids are key precursors involved in nucleotide biosynthesis, since their nitrogen molecules contribute to the synthesis of the purine ring structures^33, 34^. The accumulation of glycine and aspartic acid in elderly worms may hence suggest a downregulation of nucleotide synthesis. In this context it is important to note that in most experiments the commonly used drug 5FU—a nucleotide derivative—was added to the plates with adult worms to prevent egg hatching, which could interfere with nucleotide synthesis in these worms^35^. Interestingly, when measuring AAs in the germline deficient *glp-4*(*bn2*) mutants, their AA profile was similar to that of wild type worms, suggesting that the germline does not contribute to the observed age-related changes.

PL profiles also fluctuated during worm life history; some PL classes were abundant in early stages of life and declined with age, while others displayed an opposite pattern. When comparing the PL composition to the FA profiles, we do not see a clear correlation. This is not surprising, since PL synthesis is the result of a series of anabolic processes that result in complex lipid compositions derived from different head groups and multiple acyl chains. Overall, our findings demonstrate that the platform we developed and validated in this study can survey the metabolic changes of life history in *C. elegans*, pointing to potential links between lipid and amino acid metabolism in aging.

Many studies have shown the impact of dietary interventions at the level of gene regulation and transcription, but little is known about the changes in the metabolite profiles in *C. elegans* ^9, 12, 23, 36^. We therefore applied our platform to uncover the metabolic changes in *C. elegans* fed with three types of bacterial diets. Our findings define a clear distinction of how bacterial metabolite compositions translate to worm profiles. Worms fed a bacterial diet with a particular FA profile turned out to accumulate most of these FA species. In contrast, although the AA composition among the three bacterial diets we used was very different, feeding worms with these diets led to strikingly similar AA compositions, suggesting that AA metabolism is tightly regulated in *C. elegans*. While the regulation of FA may be less stringent, the synthesis of some FA species requires careful control. For instance, we observed that *C. elegans* contains a number of FAs that are not found in the bacterial diets, including the C18 and C20 PUFAs, demonstrating that PUFAs are mainly obtained by *de novo* elongation and desaturation of C16:0^15^. Further exploration using our metabolomics platform in combination with GC-MS should shed light on the exact regulation of this pathway. In the case of PLs we see yet another pattern associated with the different bacterial diet regimens; depending on which bacterial diet worms are exposed to, the abundance of specific PLs can vary greatly. Nevertheless, the PL composition in the worm does not directly reflect that of the bacterial strains they eat. How the bacterial diet dictates the PL composition in the worm remains to be clarified, but is likely the result of a highly regulated sequence of events involving uptake, hydrolysis, and re-synthesis, ultimately leading to the formation of these complex PL species.

In conclusion, the sensitive MS-based platform that we employed to perform worm metabolomics allowed us to discern distinct metabolic mechanisms that underpin the aging process. Further exploration of these pathways in variable genetic backgrounds and under different dietary conditions will open new avenues to understand how genetic factors and nutrition—and the combination thereof—impinge on lifespan regulation in *C. elegans*.

## Methods

### *C. elegans* strains and bacterial feeding strains

The *C. elegans* N2 (Bristol), *rrf-3*(*pk1426*) (RNAi sensitive), *glp-4*(*bn2*) and *aak2*(*ok524*) strains were obtained from the *Caenorhabditis* Genetics Center (CGC). *E. coli* OP50 was obtained from the CGC, RNAi bacterial clones are *E. coli* HT115 strains, including those to knock down *mdt-15* (R12B2.5), *fat-7* (F10D2.9), *bcat-1* (K02A4.1), which were derived from the Vidal library and were a kind gift of Dr. Yelena Budovskaya (SILS, Science Park, University of Amsterdam). *Bacillus subtilis* PY79 strain was obtained from Yelena Budovskaya (SILS, Science Park, University of Amsterdam).

### Worm growth conditions

Worms were cultured and maintained at 20 °C on nematode growth media (NGM) agar plates. For linearity and precision analyses, eggs were obtained by alkaline hypochlorite treatment of gravid adult worms grown on *E. coli* OP50, which were then seeded onto NGM plates and cultured for 2.5 days allowing development to young adults. For the RNAi validation analysis, we seeded 2000 eggs per NGMi plate (containing 2 mM IPTG and 25 mg/mL carbenicillin) with a bacterial lawn of either *E. coli* HT115 (RNAi control strain, containing an empty vector), *mdt-15* RNAi, *fat-7* RNAi or *bcat-1* RNAi bacteria. For metabolite analysis of life history traits, we collected worms cultured until selected ages. *Eggs:* approximately 150,000 eggs were collected from alkaline hypochlorite treated adult hermaphrodites; *L1 stage:* 40,000 eggs were obtained from alkaline hypochlorite treated adults and L1 worms were collected after incubating in M9 buffer overnight at 20 °C; *L2-L4 stage:* eggs were added to NGM plates seeded with *E. coli* OP50 bacteria; 20,000 worms were collected after 24 h (for L2 worms), 10,000 worms were collected after 36 h (for L3 worms), and 5000 worms were collected after 48 h (for L4 worms). *Adults:* eggs were seeded onto NGM plates containing *E. coli* OP50 bacteria and 2000 worms were collected on selected days. To prevent egg hatching, 150 µL 5 mg/mL 5FU was dropped onto 9 cm NGM plates after worms developed to L4, unless indicated otherwise. OP50 bacteria seeded onto NGM plates for the same time period were collected to measure dietary metabolite compositions. To determine the role of germline on metabolic changes during worm life history, we collected samples from *glp-4*(*bn2*) mutants and N2 worms at different ages including L3, day 1, day 3, day 5, day 7, and day 9 of adulthood. Worms were cultured at 15 °C until alkaline hypochlorite treatment and then eggs were placed onto *E. coli* OP50 seeded NGM plates and culture temperature was switched to 25 °C to allow loss of function of *glp-4* in the mutant worms. To avoid using 5FU, after both *glp-4*(*bn2*) mutants and N2 worms reached young adulthood, worms were washed from the culture plates and washed over a SEFAR NITEX^®^ 31 µM pore mesh (03–31/24) with sterile M9 buffer to filter out eggs and worms that were smaller than L4. Cleaned worm pellets were transferred onto *E. coli* OP50 seeded NGM plates on a daily basis until the desired collecting stages. To investigate the dietary intervention on metabolic changes in worms, eggs were obtained by alkaline hypochlorite treatment of N2 gravid adults grown on *E. coli* OP50. These eggs were added onto NGM plates that were seeded with *E. coli* OP50, *E. coli* HT115 or *B. subtilis* PY79 and cultured for 2.5 days to collect young adult worms.

### Fatty acid extraction and MS analysis

A synchronous population of young adult worms was washed off the plates in M9 buffer and the worm pellet was washed with dH_2_O for three times and then collected in a 2 mL Eppendorf tube and freeze-dried overnight. Dried worm pellets were stored at room temperature until use. A dry worm pellet was re-suspended in ice-cold 0.9% NaCl solution (250 µL). Worms were homogenized with a 5 mm steel bead using a TissueLyser II (Qiagen) for 2 × 2.5 min at frequency of 30 times/sec, followed by a tip sonication (energy level: 40 joule; output: 8 watts) for two times on ice water. Protein quantification was performed with BCA assay.

Fatty acids (FAs) were extracted from worm lysate based on the procedure described previously^18^. Worm lysate (up to 150 µg protein) was transferred in a 4 mL FA-free glass vial, and 1 mL of freshly prepared 100% acetonitrile (ACN)/37% hydrochloric acid (HCl) (4:1, v/v) was added to the lysate, together with deuterium-labeled internal standards (d5-C18:0 (5.04 nmol), d4-C24:0 (2.52 nmol), and d4-C26:0 (0.25 nmol)). FA samples were hydrolyzed by incubating at 90 °C for 2 h. After the vials cooled down to room temperature, 2 mL of hexane was added, and samples were mixed by vortexing for 5 sec followed by a centrifugation step at 1000 *g* for 1 min. The upper layer was transferred to a FA-free glass tube and evaporated at 30 °C under a stream of nitrogen. FA residues were dissolved in 150 µL chloroform-methanol-water (50:45:5, v/v/v) solution containing 0.0025% aqueous ammonia, and then transferred to a Gilson vial for ESI-MS analysis.

The MS system consisted of an Acquity UPLC Binary Solvent manager (Waters, Milford MA) and an Acquity UPLC sample manager connected to a Quattro Premier XE mass spectrometer (Waters, Milford MA), used in the negative ESI mode^18^.

For calculation of FA concentrations, a five-point calibration curve was made for C18:0, C24:0 and C26:0 (analytes). Of a calibration mixture containing different types of FA species, 0, 25, 50, 100, and 200 µL was added to 50 µL internal standard containing d5-C18:0 (100.8 µmol/L), d4-C24:0 (50.29 µmol/L), and d4-C26:0 (5.06 µmol/L). Samples were extracted and analyzed as described above. For each FA analyte, the input concentration (in nmol) was plotted against the ratio of the peak height of the analyte to the peak height of its corresponding internal standard. This standard curve was used to calculate the concentrations of FA analytes in the samples.

### Amino acid extraction and UPLC-MS/MS analysis

The worm homogenate was prepared as for FA analysis. To extract amino acids, worm lysate was transferred to a 2 mL Eppendorf tube, and 1 mL of 80% ACN plus 20 µL of internal standard mixture (containing 68 nmol d4-alanine, 44 nmol d3-glutamate, 40 nmol d3-leucine, 28 nmol d5-phenylalanine, 34 nmol d8-valine, 34 nmol d3-methionine, 26 nmol d4-tyrosine, 22 nmol d5-tryptophan, 46 nmol d3-serine, 48 nmol d7-proline, 24 nmol d7-arginine, 28 nmol d5-glutamine, 32 nmol d4-lysine, 26 nmol ^13^C-citrulline, 28 nmol d6-ornithine, 42 nmol d10-isoleucine, and 46 nmol d3-aspartate) were added to the lysate and homogenized by vortexing. Samples were centrifuged for 10 min at 4 °C at a speed of 16,000 *g*. The supernatant was transferred to a 4 mL glass vial and evaporated under a stream of nitrogen at 40 °C. After evaporation, 220 µl of 0.01% (v/v in MQ water) heptafluorobutyric acid was added to dissolve the residue and mixed well by vortexing. Then the suspension was transferred to a Gilson vial for HPLC-MS/MS analysis as described^19, 20, 37^.

Liquid chromatography was performed at 50 °C using a Acquity UPLC BEH C18, 1.7 µm, 2.1 × 100 mm column (Waters, Milford MA) and the injected volume was 10 µL. Mass spectrometry experiments were performed using a Micromass Quattro Premier XE Tandem Mass Spectrometer (waters, Milford, MA). The mass spectrometer was used in the multiple reaction monitoring mode (MRM) in the ESI-positive mode.

For the calculation of AA concentrations, a 50 µL standard mixture containing all amino acids with a concentration of 250 µmol/L was added to the internal standard (20 µL, same composition as mentioned above) and analyzed as described above.

### Phospholipid extraction and HPLC/MS analysis

A synchronous population of young adult worms was washed off the plates in M9 buffer, the worm pellet was washed with dH_2_O for three times and then collected in a 2 mL Eppendorf tube. The freshly collected pellet was then either immediately used for PL extraction, or flash frozen in liquid nitrogen and stored at −80 °C until measurement time, to avoid hydrolysis. Phospholipids were extracted using a one-phase extraction. Specifically, we added a defined amount of internal standards (0.1 nmol CL(14:0)_4_, 0.2 nmol BMP(14:0)_2_, 2.0 nmol PC(14:0)_2_, 0.1 nmol PG(14:0)_2_, 5.0 nmol PS(14:0)_2_, 0.5 nmol PE(14:0)_2_, 0.5 nmol PA(14:0)_2_, 2.0 nmol SM(12:0), 0.02 nmol LPG(14:0), 0.1 nmol LPE(14:0), 0.5 nmol LPC(14:0), 0.1 nmol LPA(14:0) (Avanti Polar Lipids, Alabaster, AL) dissolved in 120 µL of chloroform/methanol (1:1, v/v)), and 1.5 mL of chloroform/methanol (1:1, v/v). Subsequently, the mixture was sonicated in a water bath for 5 min, followed by centrifugation at 16,000 *g* for 5 min. The supernatant (organic layer) was then transferred to a glass vial and evaporated under nitrogen stream at 45 °C, while the protein pellet was dried and kept for quantification. Subsequently, the residue was dissolved in 150 µL of chloroform/methanol (9:1, v/v), and 10 µL of the solution was injected into the high-performance liquid chromatography-mass spectrometry (HPLC-MS) system. For protein correction, the dried pellet was dissolved in 0.2 M NaOH and protein concentrations were determined using the BCA assay.

The HPLC system consisted of a Ultimate 3000 binary (U)HPLC pump, a vacuum degasser, a column temperature controller, and an autosampler (Thermo Scientific, Waltham, MA, USA). The column temperature was maintained at 25 °C. The lipid extract was injected onto a LiChrospher 2* 250-mm silica-60 column, 5 µm particle diameter (Merck, Darmstadt, Germany). The phospholipids were separated from interfering compounds by a gradient between solution B (chloroform/methanol, 97:3, v/v) and solution A (methanol/water, 85:15, v/v). Solutions A and B contained 5 and 0.2 ml of 25% (v/v) aqueous ammonia per liter of eluent, respectively. The gradient (0.3 ml/min) was as follows: 0–1 min 10%A, 1–4 min, 10%A–20%A, 4–12 min 20%A–85% A; 12–12.1 min, 85%A–100% A; 12.1–14.0 min, 100% A, 14–14.1 min, 100%A–10%A and 14.1–15 min, equilibration with 10% A. All gradient steps were linear. A Q Exactive Plus mass spectrometer (Thermo Scientific) was used in the negative and positive electrospray ionization mode. Nitrogen was used as the nebulizing gas. The spray voltage used was 2500 V, and the capillary temperature was 256 °C. S-lens RF level: 50, Auxilary gas: 11, Auxiliary gas temperature 300 °C, Sheath gas: 48, Sweep cone gas: 2. In both negative and positive mode, mass spectra of phospholipid molecular species were obtained by continuous scanning from m/z 150 to m/z 2000 with a resolution of 280000.

For quantification of PL data, the raw HPLC/MS data were converted to mzXML format using MSConvert^38^. The dataset was processed using a semi-automated metabolomics pipeline written in the R programming language (http://www.r-project.org). In brief, it consisted of the following five steps: (1) pre-processing using the R package XCMS, (2) identification of metabolites, (3) isotope correction, (4) normalization and scaling and (5) statistical analysis.

### Metabolomics validation

For linearity analysis of FA and AA, metabolites were extracted from increasing amounts of worm protein lysate (0–250 µg for FA and 0–150 µg for AA), and PL were extracted from an increasing number of worms (0, 500, 1000, 2000 and 4000 worms) that was subsequently corrected for protein amount. Precision of FA and AA analysis was examined in several manners including MS variation, intra-assay variation and inter-assay variation. MS variation was determined by measuring FAs or AAs in the same samples and injected 21 times to the MS apparatus on the same day. The intra-assay (within day) variation was determined by measuring FAs or AAs in 12 identical samples that were extracted separately but measured within the same analytical run. The inter-assay (day-to-day) variation was assessed in several aliquots of the same dry worm pellet, which were extracted on different days and measured in different analytical runs. The limit of detection (LOD) and limit of quantification (LOQ) were defined as 3 times and 10 times the detected noise levels respectively.

### Bioinformatics

Heat maps were created using the R package ‘gplots’. Colors in the heat-maps reflect the relative metabolite abundance level with red corresponding to higher and blue to lower levels than the mean abundance value per metabolite. For PLs, prior to heat map construction we first eliminated the 5% lowest abundant species and then performed principal component analysis (PCA) using the R package ‘stats’, with which metabolites were ranked according to their contribution to principal components 1 and 2. The most contributing PL species were selected for heat map visualization.

## Electronic supplementary material

Supplementary information

Supplementary Table S1
