# Supplementary material for: A sensitive mass spectrometry platform identifies metabolic changes of life history traits in C. elegans
Source: Sci Rep. 2017 May 25;7:2408. doi: 10.1038/s41598-017-02539-w (PMC5445081; doi:10.1038/s41598-017-02539-w)
Supplement: Supplementary file 1 — Supplementary information [file 41598_2017_2539_MOESM1_ESM.pdf]

Supplementary Information

A sensitive mass spectrometry platform identifies metabolic changes of life history traits in *C. elegans*

Arwen W. Gao[1]*, Iliana A. Chatzispyrou[1]*, Rashmi Kamble[1], Yasmine J. Liu[1], Katharina Herzog[1], Reuben L. Smith[1], Henk van Lenthe[1], Martin A. T. Vervaart[1], Arno van Cruchten[1], Angela C. Luyf[2], Antoine van Kampen[2], Mia L. Pras-Raves[1,2], Frédéric M. Vaz[1], Riekelt H. Houtkooper[1#]

[1]Laboratory Genetic Metabolic Diseases; [2]Department of Clinical Epidemiology, Biostatistics and Bioinformatics, Academic Medical Center, 1105 AZ Amsterdam, The Netherlands.

*Equal contribution

#Correspondence: r.h.houtkooper@amc.nl

Supplementary Figures

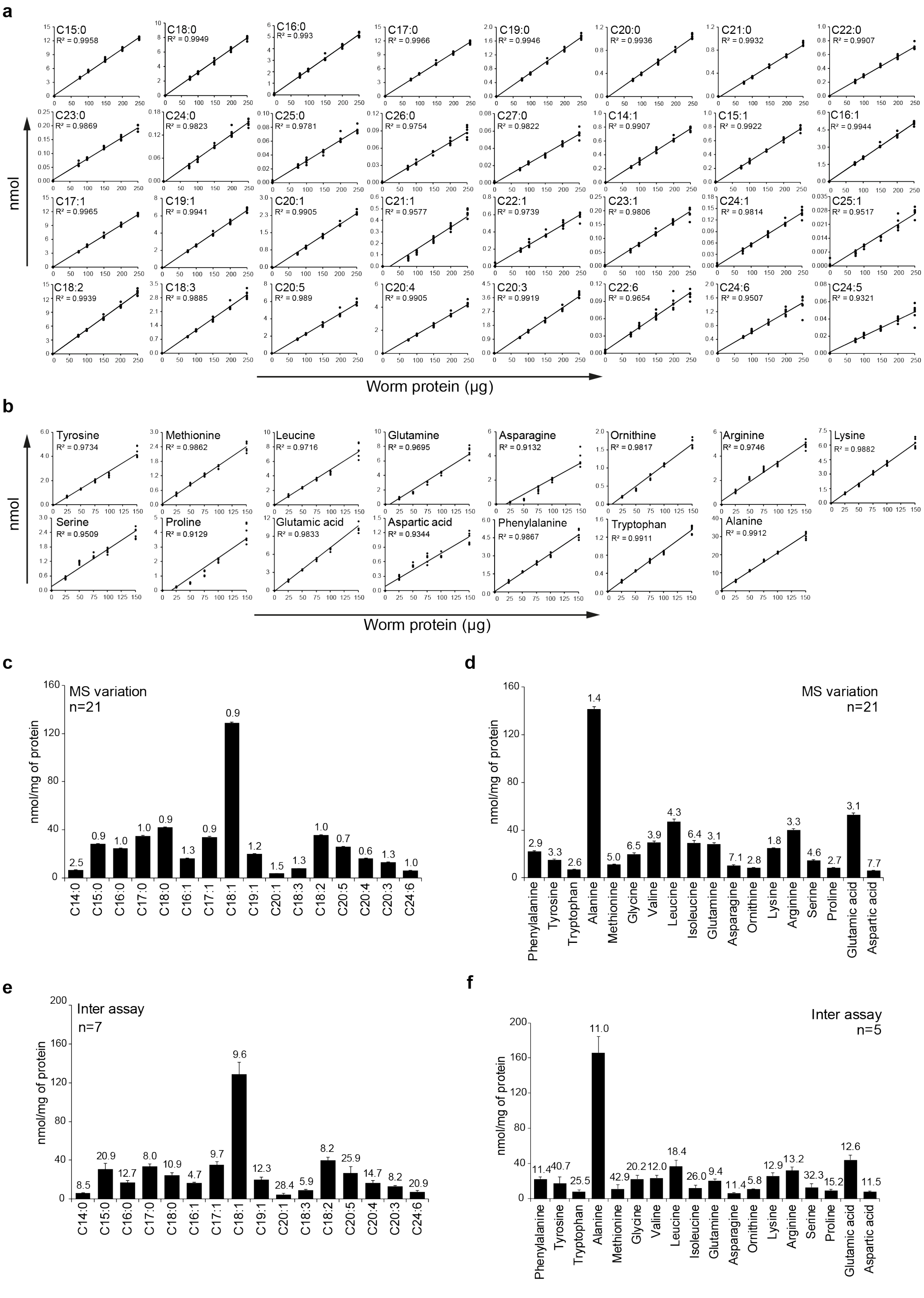

Supplementary Figure S1. Linearity and precision of fatty acid (FA) and amino acid (AA) analysis in *C. elegans*. Related to Figure 1.

(**a**) Linear response between FA levels and amount of extracted worm protein lysate (0-250 µg). A total of 35 FAs were above limit of quantification (LOQ).

(**b**) Linear response between AAs levels and amount of extracted worm protein lysate (0-150 µg). A total of 18 AA species were detectable and above LOQ.

(**c-d**) Mass spectrometry (MS) variation for FAs (A) and AAs (B) was determined by injecting the same sample 21 times into the MS.

(**e-f**) Inter-assay (day-to-day variation) variation was determined by extracting and measuring the same biological samples on different days.

Bar graphs are expressed as mean±SD. Values on top of the bars indicate the coefficient of variation.

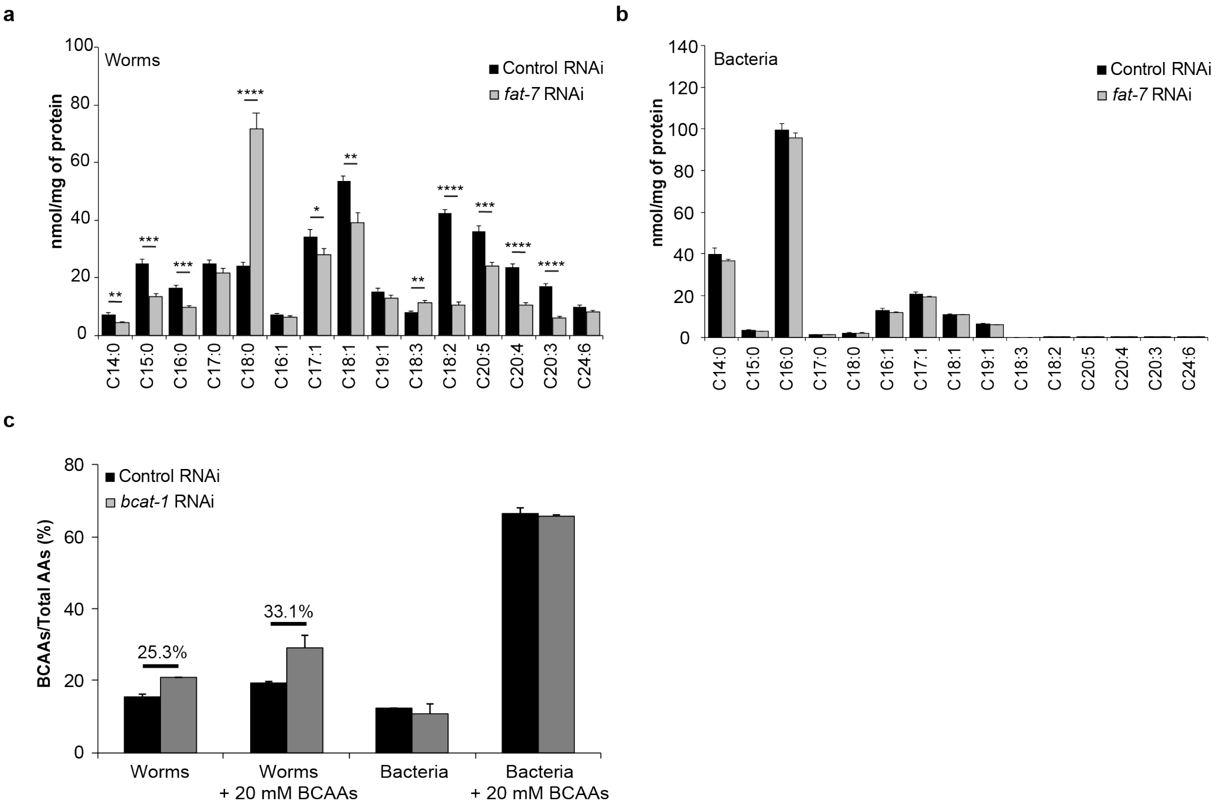

Supplementary Figure S2. Validation of FA analysis with worms deficient in lipid metabolism. Related to Figure 2.

(**a**) Worms that are deficient in *fat-7* (stearoyl-CoA desaturase) accumulated C18:0 and displayed decreased levels of C18:1 and most PUFAs.

(**b**) FA composition is similar between *fat-7* RNAi bacteria and the control RNAi bacteria. PUFAs were not detected in both RNAi bacterial strains.

(**c**) Validation of AA analysis with worms deficient in branched-chain amino acid (BCAA) metabolism. Ratio of BCAAs over total AAs was 25.3% higher in *bcat-1* RNAi worms compared to the controls. When supplementing 20 mM BCAAs in the culture medium, the ratio of BCAAs over total AAs was 19.7% higher in the control worms that grown on 20 mM BCAAs supplemented plates compared to the regular, non-supplemented plates. We found a greater accumulation of BCAAs in the *bcat-1* RNAi worms, in which the ratio of BCAAs/total AAs was 33.1% higher than the controls. There was no significant change in the ratio of BCAAs/total AAs detected between the *bcat-1* RNAi and control RNAi bacteria.

Bar graphs are expressed as mean+SD, Significance was calculated using Student's t-test. * $p \leq 0.05$; ** $p \leq 0.01$; *** $p \leq 0.001$; **** $p \leq 0.0001$

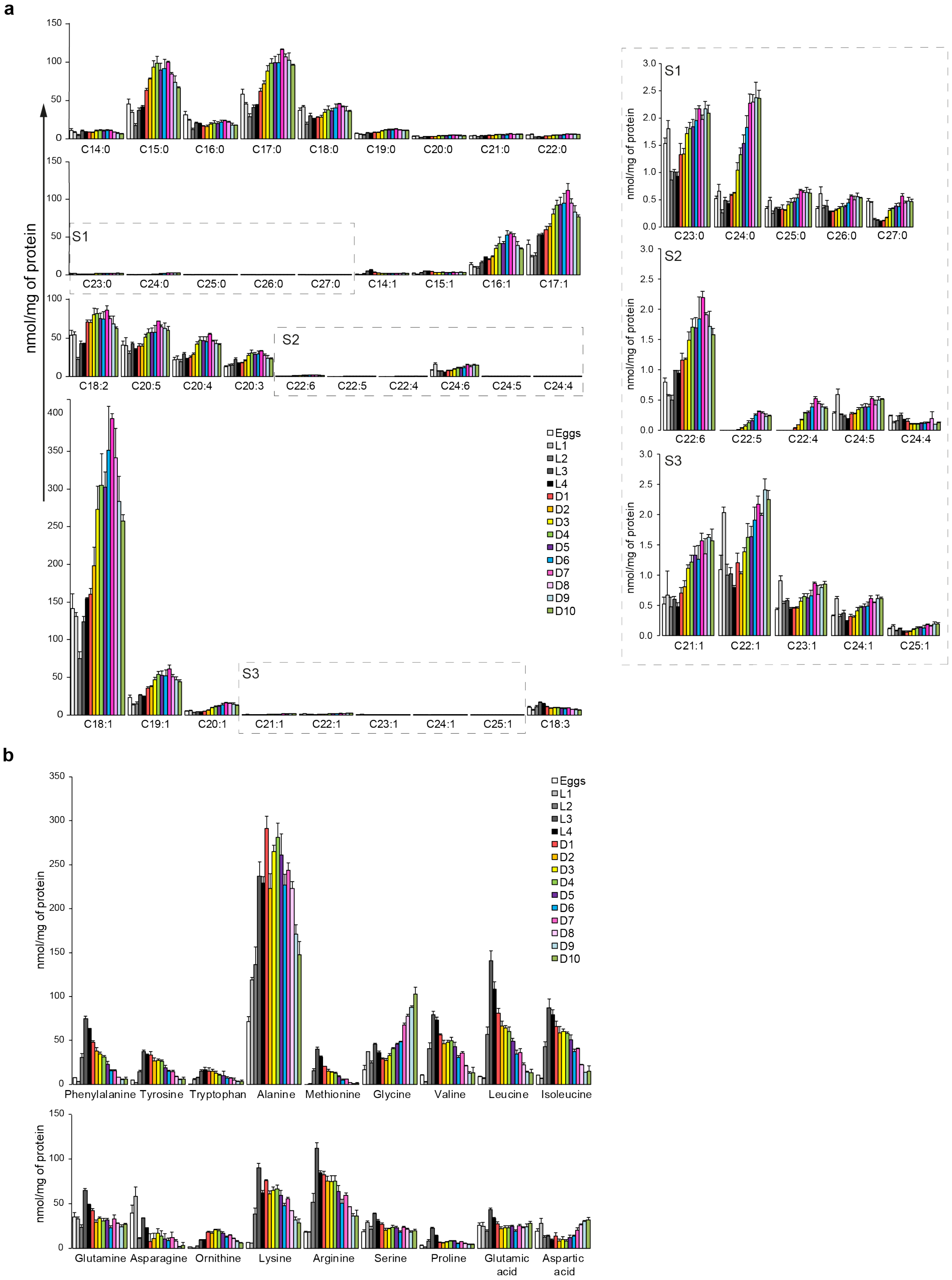

Supplementary Figure S3. Metabolic changes of life history traits in *C. elegans.* Related to Figure 4.

(**a**) 35 FAs were above LOQ over the course of worm life history. Profiles of FAs with low abundance are shown in the zoomed figures shown in the dashed boxes (S1-S3). Bar graphs are expressed as mean+SD

(**b**) Most AA species reached the highest abundance during the later stages of development and decreased in the adulthood. The abundances of aspartic acid and glycine remained at low level in the early age and significant increased in later age. Bar graphs are expressed as mean+SD

a

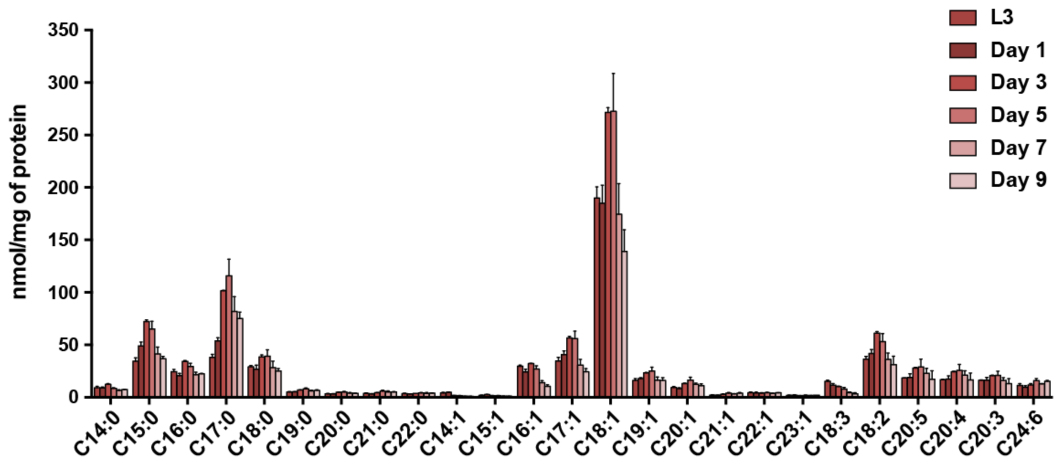

b

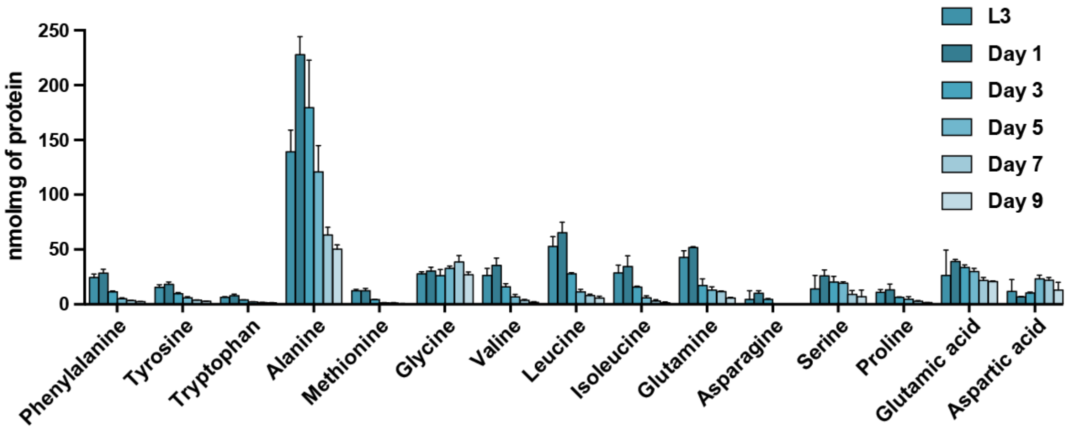

Figure S4. Metabolite profile of N2 worms cultured at 25°C and without 5FU. Related to Figure 6.

Since *glp-4(bn2)* mutants are temperature sensitive and grown without 5FU, we cultured N2 worms under the same condition (before bleaching: 15°C; after bleaching: 25°C) and collected worms at late larval stage L3, reproductive phase day 1, day 3 and day 5 of adulthood, and aging stage day 7 and day 9.

(**a**) Most abundant FAs increased with age with a peak at day 5 of adulthood, and slightly decreased in the post-reproductive phase. The highest abundance of C18:3 was observed in L3 worms and then declined with age.

(**b**) Most AAs reached a peak level at L3 and early adulthood, and declined with age, while aspartic acid levels increased, and glycine remained stable.

a

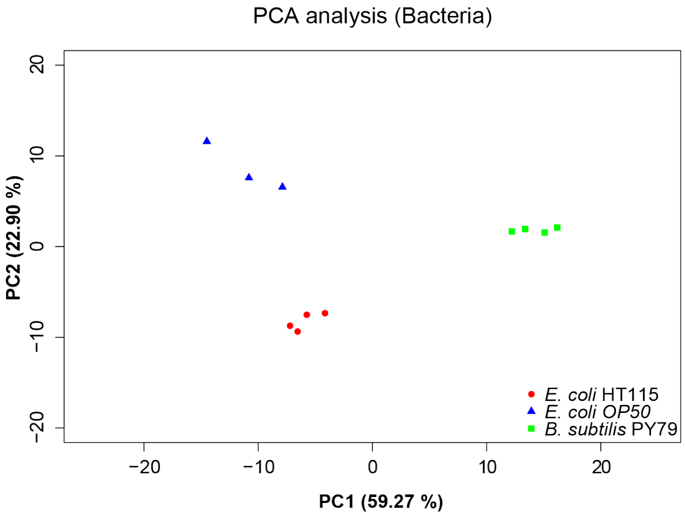

b

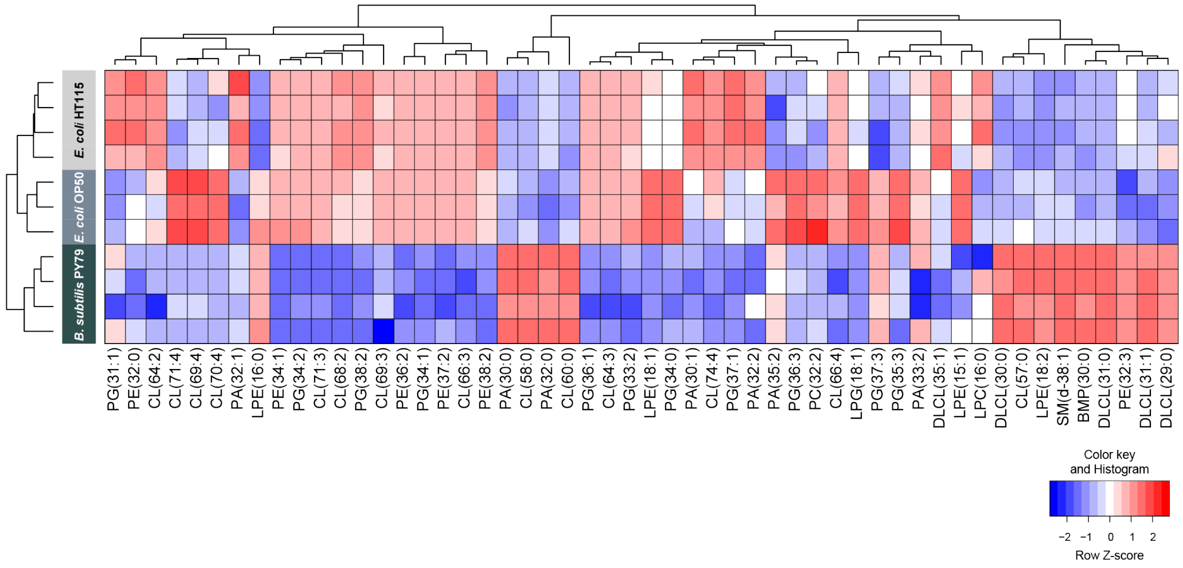

Supplementary Figure S5. Phospholipid (PL) profiles in *E. coli* OP50, *E. coli* HT115 and *B. subtilis* PY79 bacterial diets. Related to Figure 8.

(**a**) Principal Component Analysis (PCA) score plot showing group separation based on PL profiles in the three bacterial strains. *B. subtilis* PY79 was clearly separated from the *E. coli* strains, and a distinctive separation was also evident between the two *E. coli* strains.

(**b**) Unsupervised hierarchical clustering of PL species in the three bacterial diets. The top metabolites contributing to the PCA separation (**a**) are shown. All three groups of bacteria can be distinguished based on these species. Data were log2-normalised prior to creation of the heat map.
